# Tonic GABA_A_ conductance decreases membrane time constant and increases EPSP-spike precision in hippocampal pyramidal neurons

**DOI:** 10.3389/fncir.2013.00205

**Published:** 2013-12-25

**Authors:** Agnieszka I. Wlodarczyk, Chun Xu, Inseon Song, Maxim Doronin, Yu-Wei Wu, Matthew C. Walker, Alexey Semyanov

**Affiliations:** ^1^Department of Clinical and Experimental Epilepsy, UCL Institute of NeurologyLondon, UK; ^2^RIKEN Brain Science InstituteWako-shi, Japan; ^3^Department of Neurodynamics and Neurobiology, University of Nizhny NovgorodNizhny Novgorod, Russia

**Keywords:** spike jitter, EPSP-spike precision, tonic conductance, GABA, hippocampus

## Abstract

Because of a complex dendritic structure, pyramidal neurons have a large membrane surface relative to other cells and so a large electrical capacitance and a large membrane time constant (τ_m_). This results in slow depolarizations in response to excitatory synaptic inputs, and consequently increased and variable action potential latencies, which may be computationally undesirable. Tonic activation of GABA_A_ receptors increases membrane conductance and thus regulates neuronal excitability by shunting inhibition. In addition, tonic increases in membrane conductance decrease the membrane time constant (τ_m_), and improve the temporal fidelity of neuronal firing. Here we performed whole-cell current clamp recordings from hippocampal CA1 pyramidal neurons and found that bath application of 10μM GABA indeed decreases τ_m_ in these cells. GABA also decreased first spike latency and jitter (standard deviation of the latency) produced by current injection of 2 rheobases (500 ms). However, when larger current injections (3–6 rheobases) were used, GABA produced no significant effect on spike jitter, which was low. Using mathematical modeling we demonstrate that the tonic GABA_A_ conductance decreases rise time, decay time and half-width of EPSPs in pyramidal neurons. A similar effect was observed on EPSP/IPSP pairs produced by stimulation of Schaffer collaterals: the EPSP part of the response became shorter after application of GABA. Consistent with the current injection data, a significant decrease in spike latency and jitter was obtained in cell attached recordings only at near-threshold stimulation (50% success rate, S_50_). When stimulation was increased to 2- or 3- times S_50_, GABA significantly affected neither spike latency nor spike jitter. Our results suggest that a decrease in τ_m_ associated with elevations in ambient GABA can improve EPSP-spike precision at near-threshold synaptic inputs.

## Introduction

Precision of spike timing in relationship to an EPSP is computationally important, permitting more accurate data transfer (London et al., 2002). However, the large capacitance of neurons results in slow EPSPs despite rapid changes in excitatory conductances. IPSPs by curtailing the EPSPs improve spike precision (Pouille and Scanziani, 2001; Pavlov et al., 2011). There is, however, a slower form of inhibition due to tonic GABA_A_ conductances, which are produced by ambient GABA acting on extrasynaptic GABA_A_ receptors (Semyanov et al., 2004; Brickley and Mody, 2012), and possibly spontaneous openings of GABA_A_ receptors (McCartney et al., 2007; Wlodarczyk et al., 2013). Ambient GABA originates from various sources including neurotransmitter spillover (Glykys and Mody, 2007; Song et al., 2013), release by astrocytes (Bowery et al., 1976; Angulo et al., 2008; Heja et al., 2009; Lee et al., 2010)and non-synaptic release by neurons (Zilberter et al., 1999; Wu et al., 2007). The levels of ambient GABA are efficiently regulated by GABA uptake (Keros and Hablitz, 2005). Distribution of GABA transporters in the tissue may be responsible for regional differences in ambient GABA concentrations (Engel et al., 1998; Semyanov et al., 2003). Moreover, different transporter types have been shown to regulate extrasynaptic GABA originating from different sources (Kersante et al., 2013; Song et al., 2013).

Tonic activation of GABA_A_ receptors affects neurons through two mechanisms: membrane polarization (depolarization or hyperpolarization) and an increase in membrane conductance. In the adult brain both hyperpolarizing and depolarizing actions of GABA have been described depending on the neuron type (Gulledge and Stuart, 2003; Glickfeld et al., 2009; Song et al., 2011; Chiang et al., 2012). The increase in membrane conductance associated with activation of GABA_A_ receptors commonly referred as shunting inhibition. Similar to polarization the shunting inhibition affects neuronal input-output characteristics in cell type specific manner. For example, in cerebellar granular cells tonic conductance changes the gain (Mitchell and Silver, 2003), while in CA1 pyramidal neurons produces offset without affecting the gain (Pavlov et al., 2009). In addition to shunting, the tonic GABA_A_ conductance should also affect cell computation by changing τ_m_, membrane time constant (Rall, 1969; Jack and Redman, 1971). However this action of tonic GABA_A_ conductance has not been systematically investigated. Here we determined how elevation in extracellular GABA affects τ_m_, and how this change in τ_m_ combined with shunting inhibition is translated to the spike latency and jitter in response to depolarizing current injections and EPSPs.

## Methods

### Hippocampal slice preparation

Transverse hippocampal slices (300μm thick) were used for electrophysiological recordings. Slices were prepared from 3 to 4 weeks-old male Sprague Dawley rats. Animals were killed by an overdose of isoflurane according to the United Kingdom Animals (Scientific Procedures) Act of 1986. After decapitation, brains were rapidly removed and dissected, and hippocampi were sliced with a Leica VT1200S vibratome in ice-cold sucrose-based solution containing the following (mM): 70 sucrose, 80 NaCl, 2.5 KCl, 7 MgCl_2_, 0.5 CaCl_2_, 25 NaHCO_3_, 1.25 NaH_2_PO_4_, 22 glucose, equilibrated with 95% O_2_ plus 5% CO_2_, pH 7.4, 315-330 mOsm. Slices were maintained in continuously oxygenated sucrose-free storage solution at 33°C for 15 min, equilibrated to a room temperature for 15 min and then placed to recover in continuously oxygenated humidified interface chamber at room temperature for at least 1 h prior to recording. After recovering slices were transferred into recording chamber. The perfusion and storage medium contained (mM): 119 NaCl, 2.5 KCl, 1.3 MgSO_4_, 2.5 CaCl_2_, 26.2 NaHCO_3_, 1 NaH_2_PO_4_, 22 glucose and was gassed with 95% O_2_ and 5% CO_2_, pH 7.4; 290–298 mOsm. Recordings were performed at 32–34°C.

### *in vitro* electrophysiology

Visualized current clamp recordings from the CA1 pyramidal neurons were performed using infrared DIC imaging system. The resting membrane potential was maintained at −70 mV in current clamp by constant current injection (i.e., “manual voltage clamp”) in the control and was adjusted to the same value after application of 10μM GABA. CGP 52432 (1μM) was routinely added to the perfusion solution to avoid activation of GABA_B_ receptor by added GABA, except when it is stated otherwise. The whole-cell pipette resistance was 5–6 MΩ.

Square current steps of 70, 80, 90, 100, and 110 pA (500 ms) were injected via the recording pipette to estimate voltage dependence of GABA effect on the membrane time constant. Interval between recordings was 20 s. The intracellular pipette solution contained (in mM): 140 K-gluconate, 10 KOH-HEPES, 0.2 KOH-EGTA, 8 NaCl, 10 Na-phosphocreatine, 2 Mg-ATP, 0.3 Na-GTP, and 5 QX-314Br (intracellular blocker of Na^+^ channels to prevent cell spiking). To avoid rebound responses by the hyperpolarization-activated *h*-current the experiments with depolarizing current injections were performed in the presence of the *h*-channel blocker ZD7288 (15μM).

Action potentials (APs) were elicited by depolarizing square current steps of an amplitude 2, 3, 4, 5, and 6 rheobases (I_R_, 500 ms) or by depolarizing ramps of 20, 100, 200, 300, and 500 ms (from 0 to 4I_R_). To obtain the rheobase, we used repeated 500 ms square current injections with 10 pA step increases. Once the threshold was reached, we did fine adjustments with 1 pA steps. Experiments in which rheobase changed were discontinued. The intracellular pipette solution was the same as above but with omitted QX-314Br. Analyses of the first spike was used to obtain values of latency, jitter and the AP threshold.

Parameters of EPSP/IPSP waveform were measured in response to Schaffer collaterals stimulation delivered with extracellular biopolar stainless steel electrode placed in the *stratum radiatum* at least 300μm away from the recording site. 5 stimulation strengths were used in each cell to obtain the EPSP peaks ranging between 10 to 40 mV. Schaffer collaterals stimulation was also used to trigger action currents which were recorded in cell-attached mode. First, the amplitude of the stimulating current was adjusted to obtain ~50% spike probability (S_50_), and then the amplitude was increased 2 and 3 times of S_50_. After GABA application S_50_ was established again to compensate for EPSP shunting. These experiments were performed using patch pipette (8–10 MΩ) filled with ACSF in voltage clamp mode with voltage adjusted so no current was injected (holding current = 0pA).

### Data acquisition and analysis

Recordings were obtained using a MultiClamp 700B amplifier, filtered at 4 kHz and digitized at 10 kHz when APs were not measured. Otherwise the recordings were filtered at 24 kHz and digitized at 50 kHz. Homemade LabView software or WinWCP (supplied free of charge to academic users by Dr. John Dempster, University of Strathclyde, UK) were used for data acquisition. pClamp (Molecular Devices) and OriginPro 8.1 (OriginLab) for off-line data analysis.

τ_m_ and EPSP decay time constant were estimated from single exponential fit of voltage responses and EPSPs, respectively.

The spike threshold was obtained as the membrane voltage on the phase diagrams (Δ*V*/Δ*t* vs. *V*) at which Δ*V*/Δ*t* = 20mV/ms. Latency was defined as the time from the onset of the stimuli to the peak of the spike and was estimated from the Δ*V*/Δ*t* vs. *t* traces. Spike jitter was calculated as the standard deviation of the first spike latencies.

Data are presented as mean ± s.e.m. Differences were considered significant when *P* < 0.05. Statistical comparisons were made using one-way, two-way and repeated measure ANOVA and paired *t*-test as stated in the text.

### Modeling

Simulations were performed with the NEURON 7.1 simulation environment (Hines and Carnevale, 1997). The model was based on the realistic morphology of a CA1 hippocampal pyramidal cell modified from the study by Poirazi et al. (2003). The implemented membrane mechanisms included location-dependent R_m_ (membrane resistance) and R_a_ (axial resistance), with mixed conductances: including sodium, delay rectifier-, A-, M-type, Ca^2+^-activated potassium, and *h*-type conductances; as well as L-, R-, and T-type voltage-dependent calcium channels. The detailed gating properties of the listed channels and their subcellular distribution and density are available in the online supplemental information of the study by Poirazi et al. (2003).

Excitatory synaptic conductance was composed of AMPA and NMDA type ionotropic glutamate receptors (gAMPA and gNMDA): gAMPA was represented by a double exponential function with τ_rise_ of 1 ms, τ_decay_ of 12 ms (Otmakhova et al., 2002); gNMDA was implemented from the study by Kampa et al. (2004). Six excitatory synapses were placed on six different apical oblique dendrites within the *striatum radiatum*. For an individual excitatory synapse, gAMPA, and gNMDA were set to generate a 10 pA somatic EPSC with a physiological NMDA/AMPA charge ratio (Otmakhova et al., 2002). The outward-rectifying tonic GABAergic conductance (gGABA) was modified from the study by Pavlov et al. (2009) and used in the range of 0–3mS/cm^2^ with *E*_GABA_ = −75mV. The initial resting membrane potential of simulated neurons was −70 mV and the simulation temperature was 34°C.

In the simulations, in which the effect of tonic GABA_A_ conductance on the spike jitter was investigated, background synaptic noise was simulated by injecting a noisy conductance template (*g*_noise_) containing Poisson-distributed excitatory synaptic conductances of constant amplitude (times the unitary conductance *g*_e_ of 0.7nS) and a reversal potential of 0 mV. The time course of synaptic conductances (τ_rise_ = 0.5ms, τ_decay_ = 5ms) was used to fit evoked AMPAR-mediated EPSCs. The amplitude range and mean interevent interval of simulated background synaptic conductances (60 Hz) were chosen to mimic membrane potential variations observed *in vivo* in cortical and hippocampal pyramidal cells (Destexhe and Pare, 1999; Hahn et al., 2007). Variations of the membrane potential were measured as root mean square (RMS) noise.

## Results

**Figure 1:**
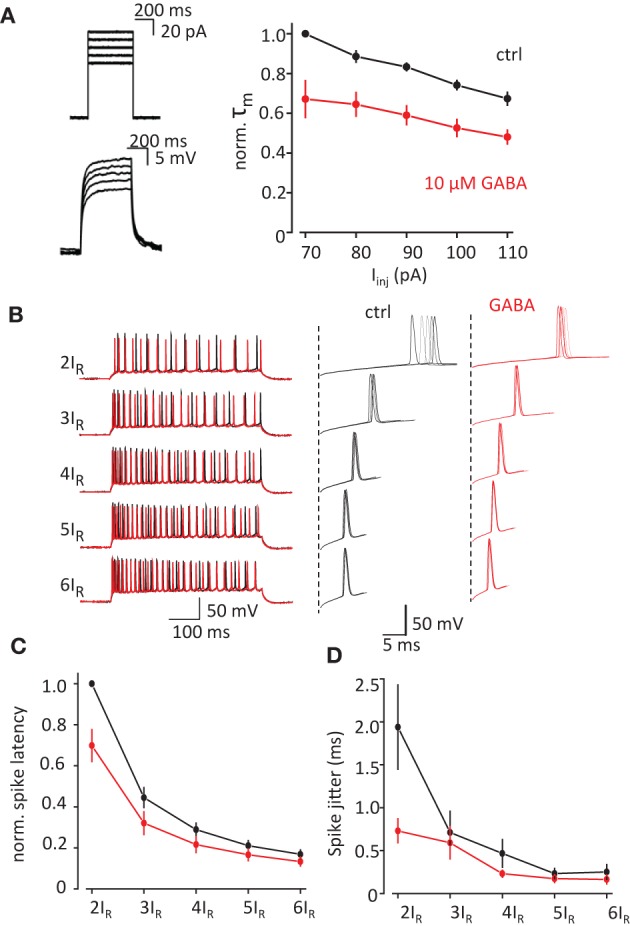
**Change in τ_m_ by exogenous GABA correlates with decrease in spike latency and spike jitter. (A)** τ_m_ measured from the timecourse of voltage change in response current injection. APs were blocked with intracellular QX-314Br. *Left*, sample traces of current injection (*above*) and corresponding voltage changes (*below*). *Right*, normalized τ_m_ in response to different current injections in control conditions (*black trace*) and in the presence of GABA (*red trace*). **(B)** APs recorded in response to current injection adjusted to 2–6 rheobases (2I_R_–6I_R_). *Left*, full traces. *Right*, zoomed first APs in the responses showing spike latency and spike jitter. Black traces—control, red traces—in the presence of GABA. **(C**,**D)** Summary data for the change in the spike latency and spike jitter by exogenous GABA. Data presented as mean ± s.e.m. (error bars). Statistical comparison is discussed in the text.

We performed current-clamp recordings in visually identified CA1 pyramidal neurons in rat hippocampal slices. τ_m_ was calculated from the exponential fit of the rise time of the voltage response to square current injection (*I*_inj_) through recording pipette (APs were blocked with intracellular QX314, Figure 1A). τ_m_ decreased with the amplitude of *I_inj_* reflecting the activation of voltage dependent conductances. 10μM GABA significantly reduced τ_m_ [*F*_(1, 3)_ = 20.0, *p* = 0.021 for decrease in τ_m_ by GABA, *F*_(4, 12)_ = 41.6, *p* < 0.001 for decrease in τ_m_ with *I*_inj_, *F*_(4, 12)_ = 2.6, *p* = 0.09 for interaction; repeated measures two-way ANOVA].

We next tested how change in τ_m_ can affect the spike latency and spike jitter in response to depolarizing current injections (500 ms, Figure 1B). To compensate for the decrease in voltage due to shunting and cell-to-cell variability we used *I*_inj_ adjusted to 2–6 rheobases (2I_R_-6I_R_) in control and then in the presence of GABA. Both spike latency and spike jitter decreased with increase in *I*_inj_ in control conditions and there further decreased by 10μM GABA (Figures 1C,D). The results were analyzed with repeated measures two-way ANOVA where *I*_inj_ and GABA were two factors affecting spike latency and spike jitter. The decrease in the latency was significant for both factors: *I*_inj_ [*F*_(4, 12)_ = 340.4 *p* < 0.001] and GABA [*F*_(1, 3)_ = 14.2 *p* = 0.03]. There was also a significant interactions between *I*_inj_ and GABA effects [*F*_(4, 12)_ = 10.032 *p* = 0.001]. (An interaction means that the response of the variable to one factor depends on the value of the other i.e., in this case the response to current injection depends upon whether GABA was there or not). Same analysis showed that the decrease in jitter was only significant for *I*_inj_ [*F*_(4, 12)_ = 15.3, *p* < 0.001] but not for GABA [*F*_(1, 3)_ = 4.7, *p* = 0.117]. There was however a significant interaction between current injection and GABA effects [*F*_(4, 12)_ = 8.87, *p* = 0.001; repeated measures two-way ANOVA, *n* = 4 cells]. These results indicate that the change in jitter and latency with current injection is altered by the addition of GABA, which results in less dependence of these on the magnitude of current injection.

**Figure 2:**
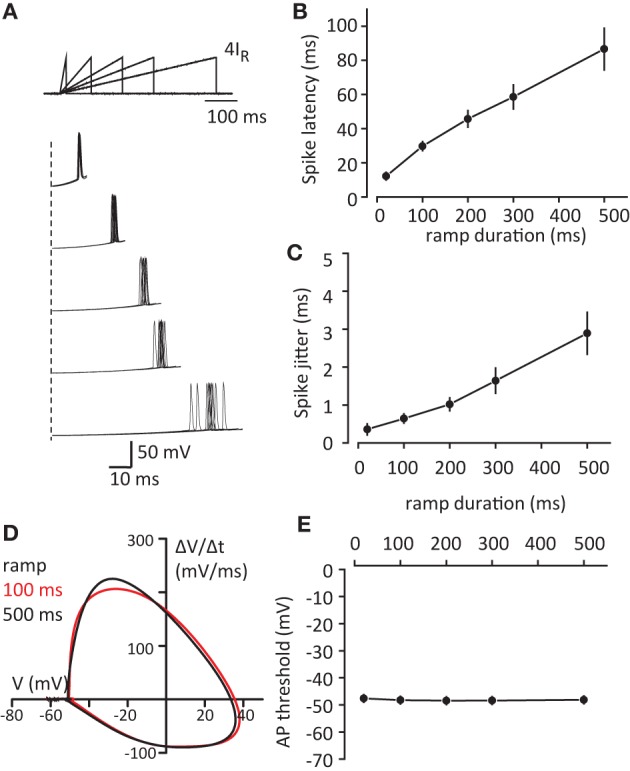
**Increase in voltage slope decreases spike latency and spike jitter. (A)** Sample traces of APs in response to current injection ramps (4IR; 10, 100, 200, 300, 500 ms). *Top*, schematic of the ramps. *Bottom*, first spikes to ramps repeated 10 times for each duration. Please note different time scales for top and bottom panels. **(B,C)** Summary data for the first spike latency and spike jitter in response to different current ramps, respectively. **(D)** Phase plot of first APs in response to 100 ms and 500 ms ramps which were used for AP threshold detection. **(E)** Summary of AP threshold for different current ramps. Data presented as mean ± s.e.m. (error bars). Statistical comparison is discussed in the text.

The observed changes in spike latency and jitter are consistent with the decrease in the rise time of voltage responses. When the voltage rise time becomes faster it also passes the AP threshold window faster. In contrast during slow voltage rises, the membrane potential will remain longer in “threshold range” allowing larger spike jitter. To test this prediction we measured spike latency and spike jitter in response to current ramps of variable slope (4I_R_ amplitude and durations from 20 to 500 ms; Figure 2A). Indeed both spike latency and spike jitter increased linearly with ramp duration [latency: *F*_(1, 5)_ = 42.2, *p* < 0.001; and jitter: *F*_(1, 5)_ = 16.1, *p* = 0.01; repeated measures one-way ANOVA, *n* = 6 cells, Figures 2B,C]. Another potential explanation is that slower ramps inactivate some sodium channels and this affects the AP threshold which could be responsible for the observed increase in spike latency and spike jitter. The AP threshold was calculated using phase plot method (Δ*V*/Δ*t* vs. *V*, Figure 2D), where threshold was obtained as membrane voltage (V) at the initial phase Δ*V*/Δ*t* = 20mV/ms. No significant difference was observed in AP threshold depending on the duration of the voltage step [*F*_(4, 20)_ = 0.039, *p* = 0.997; repeated measures one-way ANOVA, *n* = 6 cells, Figure 2E].

**Figure 3:**
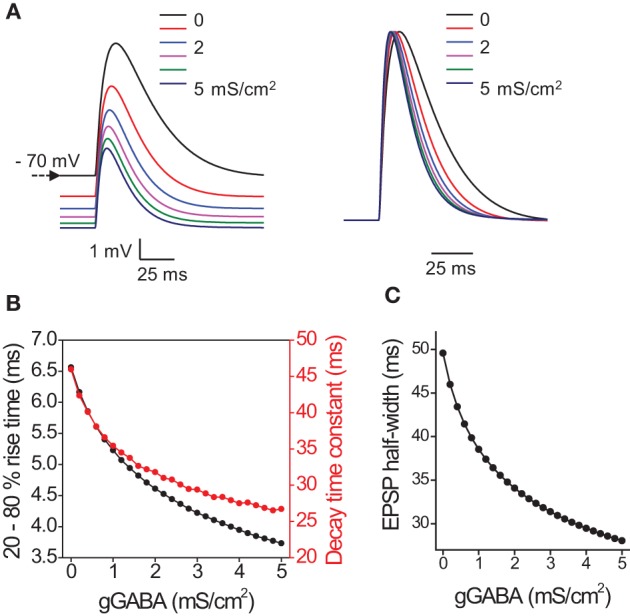
**Effect of tonic GABA_A_ conductances on the timecourse of EPSP in model pyramidal neuron. (A)** Sample EPSPs color coded for the magnitude of tonic GABA_A_ conductance (0–5 mS/cm^2^ with step 1 mS/cm^2^). *Left*, EPSPs without normalization. Note hyperpolarizing shift in membrane potential produced by tonic GABA_A_ conductance. *Right*, normalized EPSPs. Note acceleration of rise and decay times. **(B)** Summary data for the change in EPSP 20–80% rise time (black circles) and decay time constant (red circles) with tonic GABA_A_ conductance (gGABA). **(C)** Summary data for the change in EPSP half-width with gGABA.

Thus we demonstrated that tonic GABA_A_ conductance decreases the membrane time constant, which shortens the rise time of the voltage response to current injection and leads to shorter latency of the first spike and less spike jitter with low input amplitudes. The changes in tonic GABA_A_ conductance can also affect EPSP time course and precision of EPSP-spike coupling. However, an increase in ambient GABA required for a change in tonic conductance can also have a number of network effects ranging from changes in glutamate release probability (Isaacson et al., 1993) to changes in excitability of interneurons (Song et al., 2011). To estimate the isolated effect of tonic GABA_A_ conductance on EPSPs, we built a mathematical model of CA1 pyramidal neuron which received 6 synaptic inputs at different places along the dendrite, producing a multisynaptic EPSP in soma (see Methods). Then different amounts of hyperpolarizing outwardly rectifying tonic GABA_A_ conductance was introduced (*E*_GABA_ = −75mV, initial membrane potential = −70mV) (Pavlov et al., 2009). This shifted membrane potential toward more hyperpolarizing values and decreased the EPSP amplitude through shunting inhibition (Figure 3A). As expected the EPSP rise time and decay time constant also decreased in a conductance dependent manner (Figure 3B). This led to a corresponding decrease in EPSP half-width (Figure 3C).

**Figure 4:**
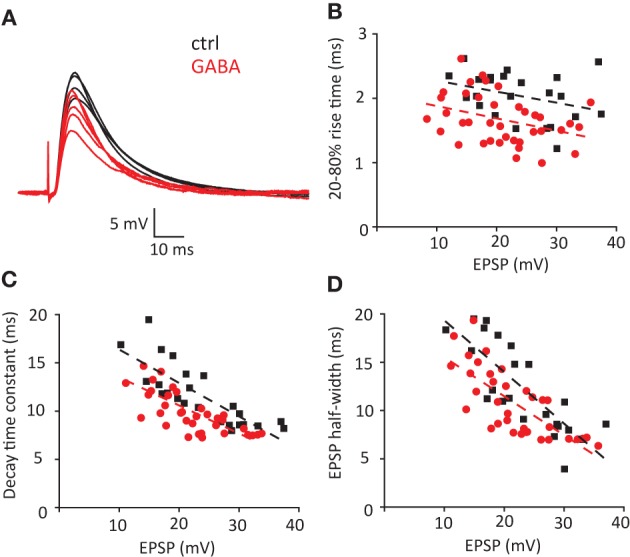
**Effect of exogenous GABA on the timecourse of EPSP/IPSP waveform triggered by stimulation of Schaffer collaterals. (A)** Current clamp sample traces recorded in control conditions (ctrl) and in the presence of GABA. 5 stimuli were delivered in each cycle of stimulation to obtain EPSP distributed in the range 10–40 mV. Same stimulation intensities were used in control and in the presence of GABA. Decrease in EPSP amplitude reflects shunting effect. **(B–D)** Individual EPSP measurements obtained in 7 cells: 20–80% rise time **(B)**, decay time constant **(C)** and half-width **(D)**. Black squares—control; red circles—GABA. Dashed lines of corresponding color—linear fits of the data.

Next we tested the effect of exogenous GABA on properties of EPSPs triggered by stimulation of Schaffer collaterals (Figure 4A). In these conditions, the EPSP time course was strongly affected by disynaptic IPSPs (Pouille and Scanziani, 2001). Five stimulus intensities were used to obtain EPSP amplitudes ranging approximately between 10 and 40 mV (APs were blocked in these experiments with intracellular QX314, *n* = 7 cells). The EPSP rise times, decay time constants and half-widths decreased with increase in EPSP amplitude. Consistent with the model and *I*_inj_ experiments, GABA decreased rise times, decay time constants and half-widths of EPSPs (Figures 4B–D). Because these experiments were performed with GABA_B_ receptors blocked, we further tested whether activation of GABA_B_ receptors can also affect EPSP time course. GABA application without GABA_B_ receptors blocked produced a similar effect (Figure S1), indicating that GABA_B_ receptors play a minimal role in this effect.

**Figure 5:**
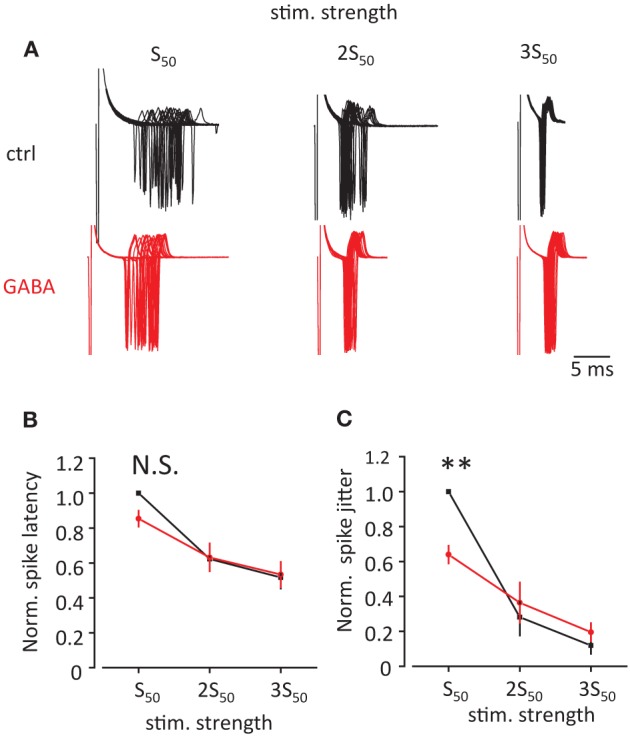
**Effect of GABA on spike latency and spike jitter in response to stimulation of Schaffer collaterals. (A)** Sample traces of cell-attached recordings in voltage clamp showing action currents triggered by stimulation of Schaffer collaterals in control conditions (black) and in the presence of GABA (red). First, the stimulation was adjusted to obtain approximately 50% success rate (S_50_). Then it was increased 2 and 3 times (2S_50_ and 3S_50_). **(B,C)** Normalized spike latency **(B)** and spike jitter **(C)** in control conditions (black) and in the presence of GABA (red). Data presented as mean ± s.e.m. (error bars). N.S. *p* > 0.05; ^**^P < 0.01; paired *t*-test.

Finally we tested how this change in EPSP/IPSP waveform produced by GABA can affect the precision of EPSP-spike coupling. We recorded action currents in cell attached mode in CA1 pyramidal neurons in response to Schaffer collateral stimulation. First the stimulation strength was adjusted to obtain action currents in approximately 50% of cases (S_50_, Figure 5A). Then the stimulus strength was increased 2 and 3 times of S_50_ (2S_50_ and 3S_50_). We observed that such an increase in stimulus strength decreased both spike latency and spike jitter. Consistent with current injections we observed that GABA application produced a decrease in spike latency and jitter only for S_50_, but not for 2S_50_ and 3S_50_ (this was however significant only for spike jitter 64 ± 5% of control, *p* = 0.004; but not for spike latency 85 ± 5% of control *p* = 0.08, *n* = 6, paired *t*-test; Figures 5B,C).

**Figure 6:**
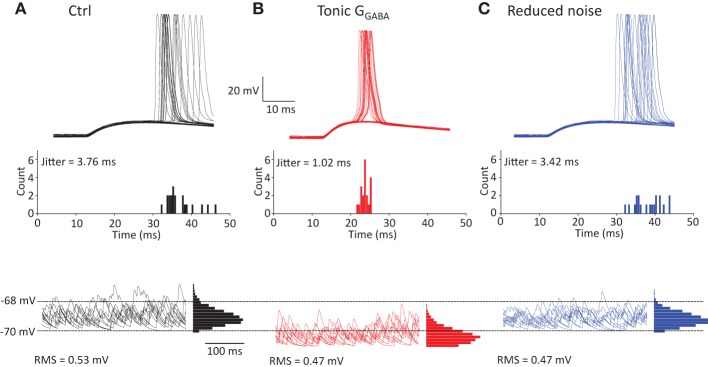
**Effect of tonic GABA_A_ conductance on spike jitter in modeled CA1 pyramidal neuron. (A)** Neuron received multisynaptic EPSP and background synaptic noise. The amplitude of EPSP was adjusted to obtain 50% spike success rate. *Top*, sample traces showing spike generation; *Middle*, histogram showing spike count at different delays from the EPSP onset; *Bottom*, synaptic noise in the neuron with corresponding all-point-histogram. **(B)** Same as at **(A)** + tonic GABA_A_ conductance, *G*_GABA_ = 1mS/cm^2^. **(C)** Same as **(A)** but RMS of synaptic background noise was reduced by the same amount as by tonic GABA_A_ conductance in **(B)**.

Although this result is consistent with accelerated EPSP/IPSP waveform because of the decrease in τ_m_, there is alternative explanation for the change in EPSP-spike coupling. Spike jitter may also result from fluctuations in membrane potential during near-threshold EPSP. These fluctuations can be reduced by tonic GABA_A_ conductance, thus reducing the spike jitter. To test this, we analyzed spike jitter in the model of CA1 pyramidal neuron receiving both near-threshold multisynaptic EPSPs and non-synchronized monosynaptic EPSPs representing background synaptic noise (Figure 6A). Coincidence of both inputs produced spikes with 50% probability, while background synaptic noise was responsible for the spike jitter. Consistent with our experimental findings, the tonic GABA_A_ conductance (1 mS/cm^2^) decreased spike jitter (Figure 6B). However, this effect was correlated with the decrease in the background synaptic noise (root-mean square, or RMS). To isolate the effect of a decrease in RMS of noise on spike jitter, we repeated the simulation without a tonic GABA_A_ conductance, but the background synaptic noise was proportionally reduced (Figure 6C). This produced a relatively small change in spike jitter suggesting that the effect of tonic GABA_A_ conductance on spike jitter is predominantly determined by the change in multisynaptic EPSP kinetics.

## Discussion

The tonic GABA_A_ conductance is relatively small in CA1 pyramidal neurons, but can increase upon ambient GABA elevation (Semyanov et al., 2004). The magnitude of tonic GABA_A_ conductance in these cells can also increase as a result of changes in subunit composition of extrasynaptic GABA_A_ receptors reported both in physiological conditions such as puberty (Shen et al., 2007) and in pathological conditions such as epilepsy (Scimemi et al., 2005). An increase in the tonic GABA_A_ conductance has been proposed as a mechanism that decreases excitability (Semyanov et al., 2003), produces neuronal offset (Pavlov et al., 2009), and impairs LTP (Shen et al., 2010) in these neurons. Now, we show that the tonic GABA_A_ conductance also affects computations in CA1 pyramidal neurons by decreasing τ_m_. A smaller τ_m_ shortens the rise time of voltage responses to current injection which correlates with a decrease in spike jitter. Because the time-course of voltage responses non-linearly depends on membrane potential (Zsiros and Hestrin, 2005), the observed change in spike jitter depends on the magnitude of the voltage response. The larger voltage response has a faster rise time which makes spike jitter less sensitive to the increase in membrane conductance produced by GABA.

Our mathematical model demonstrated that EPSP rise time, decay time and half-width are also sensitive to the change in tonic GABA_A_ conductances. We used the modeling approach to avoid potential confounding effects of ambient GABA on presynaptic glutamate release (Kullmann et al., 2005). Then we investigated the effect of ambient GABA on the time-course of EPSP/IPSP waveform produced by stimulation of Schaffer collaterals. Stronger synaptic stimulation produced significantly shorter EPSPs, which can be explained not only by the decrease in τ_m_, but also by the increased contribution of disynaptic IPSPs (Pouille and Scanziani, 2001). A decrease in EPSP half-width correlated with decreased spike latency and spike jitter. Tonic GABA_A_ conductances produced by exogenous GABA application decreased EPSP rise-time, decay-time and half-width. However, this significantly decreased spike jitter only in response to near-threshold EPSPs. Thus GABA elevation associated with network activity [e.g., during exploratory behavior (Bianchi et al., 2003)] affects coding of information which is determined by EPSP-spike precision in CA1 pyramidal neurons (Konig et al., 1996) only for certain strength of synaptic inputs. Because the tonic conductance also decreases the amplitude of EPSPs, the activation of a greater number of presynaptic fibers is required for the cell to reach the threshold, which will also reduce the spike jitter.

Notably, the regulation of the EPSP-spike precision by changes in membrane conductance is not limited to tonic activation of GABA_A_ receptors. For example, increases in *h*-conductance (*G_h_*) shorten EPSP half-duration, while decreases in *G_h_* make EPSP wider (Wu et al., 2012). This also leads to corresponding change in spike jitter in hippocampal pyramidal neurons (Gastrein et al., 2011). However, changes in *G_h_* and tonic GABA_A_ conductance have notable differences. *G_h_* change is a form of neuronal plasticity which may be triggered by activation of synaptic (increase in *G_h_*) or extrasynaptic (decrease in *G_h_*) NMDA receptors (Fan et al., 2005; Campanac et al., 2008; Wu et al., 2012) and is rather slow (minutes) process. The changes in tonic GABA_A_ conductance in addition to plasticity (changes in GABA_A_ receptors) can provide operational regulation of EPSP-spike precision. That may occur during high levels of network activity and consequent elevations of ambient GABA.

Our result is also in line with previous reports demonstrating that increases in tonic GABA_A_ conductances decrease the integration time window for coincidence detection in neurons of the chicken *nucleus laminaris* (Tang et al., 2011). Enhanced EPSP-spike precision and narrowed integration time window because of a decrease in τ_m_ can be considered as additional mechanisms responsible for the cognitive changes associated with altered levels of tonic GABA_A_ conductance which occur in physiological and pathological conditions such as the ovarian cycle (Maguire et al., 2005), puberty (Shen et al., 2010), epilepsy (Pavlov and Walker, 2013), and alcohol intake (Mody et al., 2007).

## Author contribution

Agnieszka I. Wlodarczyk, Inseon Song, Maxim Doronin performed the experiments; Agnieszka I. Wlodarczyk, Inseon Song, Maxim Doronin, Matthew C. Walker, Alexey Semyanov analyzed the data; Alexey Semyanov and Yu-Wei Wu prepared the figures; Chun Xu performed the preliminary experiments and discovered the effect of GABA on spike jitter; Yu-Wei Wu performed mathematical modeling; Alexey Semyanov and Matthew C. Walker planned the study; Alexey Semyanov wrote the manuscript; all authors commented and edited the manuscript.

### Conflict of interest statement

The authors declare that the research was conducted in the absence of any commercial or financial relationships that could be construed as a potential conflict of interest.

## Supplementary material

Supplementary Figure 1 **Effect of exogenous GABA on the timecourse of EPSP triggered by stimulation of Schaffer collaterals without blocking GABA_B_ receptors.** EPSP measurements obtained in 4 cells: 20–80% rise time (*top row*) and decay time constant (*bottom row*). Black graphs—control; red graphs—GABA.
