# Supplementary figures and images for: Tonic GABAA conductance decreases membrane time constant and increases EPSP-spike precision in hippocampal pyramidal neurons
Source: Front Neural Circuits. 2013 Dec 25;7:205. doi: 10.3389/fncir.2013.00205 (PMC3872325; doi:10.3389/fncir.2013.00205)

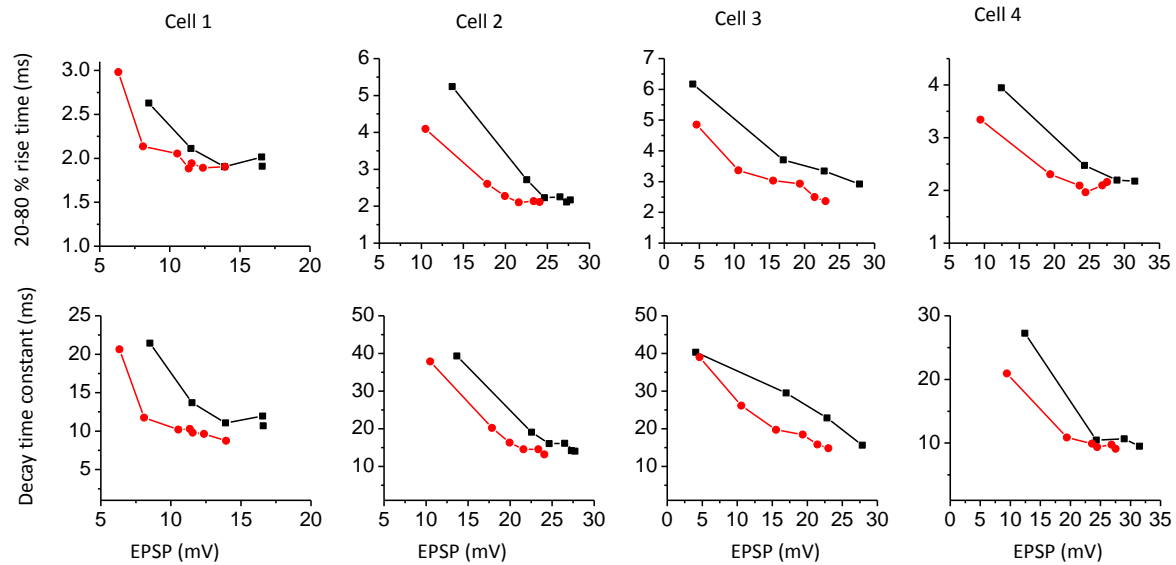

Supplement: Supplementary Figure 1 — Effect of exogenous GABA on the timecourse of EPSP triggered by stimulation of Schaffer collaterals without blocking GABAB receptors. EPSP measurements obtained in 4 cells: 20–80% rise time (top row) and decay time constant (bottom row). Black graphs—control; red graphs—GABA. [file Presentation1.PDF]
